# Surgical Complications of Primary Rhegmatogenous Retinal Detachment: A Meta-Analysis

**DOI:** 10.1371/journal.pone.0116493

**Published:** 2015-03-03

**Authors:** Zhiping Lv, Ying Li, Yongzhong Wu, Yi Qu

**Affiliations:** 1 Department of Ophthalmology, Qilu Hospital of Shandong University, No. 107, Wenhuaxi Road, Jinan 250012, China; 2 State Key Lab of Crystal Materials, Shandong University, Jinan 250100, China; Medical University Graz, AUSTRIA

## Abstract

**Background:**

To investigate the surgical complications of scleral buckling (SB) and pars plana vitrectomy (PPV) performed on primary rhegmatogenous retinal detachment (RRD) and to discover which surgical procedures bring fewer complications.

**Methods:**

An electronic literature search using the PubMed database, ISI Web of Knowledge and the Cochrane Central Register of Controlled Trials to identify randomized controlled trials and observational studies comparing SB with PPV on primary RRD. Outcome measures included intra-operative complications and early and late post-operative complications.

**Results:**

During the operation, significantly less subretinal hemorrhage occurred in the PPV group than in the SB group (OR = 4.71; 95%CI, 1.33–16.64; *p* = 0.02) and the hypotony incidence was significantly higher in the SB group (OR = 18.24; 95%CI, 2.37–140.44; *p* = 0.005); however, the occurrence of iatrogenic breaks was significantly lower in the SB group (OR = 0.05; 95%CI, 0.01–0.21; *p*<0.0001). In the early stage of post-operation, significantly higher incidence of choroidal detachment was identified in the SB group than in the PPV group (OR = 10.19; 95%CI, 2.36–44.09; *p* = 0.002); patients undergoing SB had significantly higher odds of residual subretinal fluid (OR = 14.71; 95%CI, 1.84–117.32; *p* = 0.01); the occurrence of high intraocular pressure was significantly lower in the SB group (OR = 0.46; 95%CI, 0.23–0.89; *p* = 0.02); and no significant difference was shown in the incidence of epithelia defect (*p* = 0.37) between the two groups. In the late stage of post-operation, the incidence of diplopia/extraocular muscle dysfunction was significantly higher in the SB group (OR = 4.04; 95%CI, 1.30–12.52; *p* = 0.02); and significantly less cataract was observed in the SB group (OR = 0.20; 95%CI, 0.14–0.30; *p*<0.00001); no significant difference was found in the incidences of cystoid macular edema (*p* = 0.65), macular pucker (*p* = 0.52), post-operative proliferative vitreoretinopathy (*p* = 0.73) and epiretinal membrane (*p* = 0.47) in other late post-operative complications.

**Conclusions:**

This meta-analysis suggests that PPV could be considered as potential surgical management on primary RRD.

## Introduction

Rhegmatogenous retinal detachment (RRD) is characterized by a breach in the neurosensory retina with seepage of fluid into the subretinal space which is the most common form of retinal detachment[1]. If left untreated, most patients will progress to complete loss of vision. The treatment strategy on the primary RRD has undergone considerable changes over recent decades[2]. The choice of surgical method, along with its technical specifications, varies significantly between surgeons and centers. Scleral buckling (SB) and pars plana vitrectomy (PPV) are the most commonly used procedures because of their anatomical success rates are over 90% respectively[3,4]. SB is usually considered as the “golden standard” for cases with single breaks and limited retinal detachments[5], PPV is the treatment of choice for cases with large tears, breaks at the posterior pole, proliferative vitreoretinopathy (PVR) grade-C or more, or vitreous hemorrhage[6,7]. A systematic review conformed that SB provided superior post-operative outcomes than PPV on primary RRD[8]; however, a meta-analysis using data from retrospective studies indicated that PPV combined with or without SB achieved better outcome than SB on such cases[9]. No consensus can be reached on the preferred surgical approach for the management of primary RRD. In this meta-analysis we evaluated the surgical complications occurred during the operation, early or late stage after operation between SB and PPV on primary RRD, attempting to find the preferable therapeutic option.

## Materials and Methods

### Literature search

**Fig 1:**
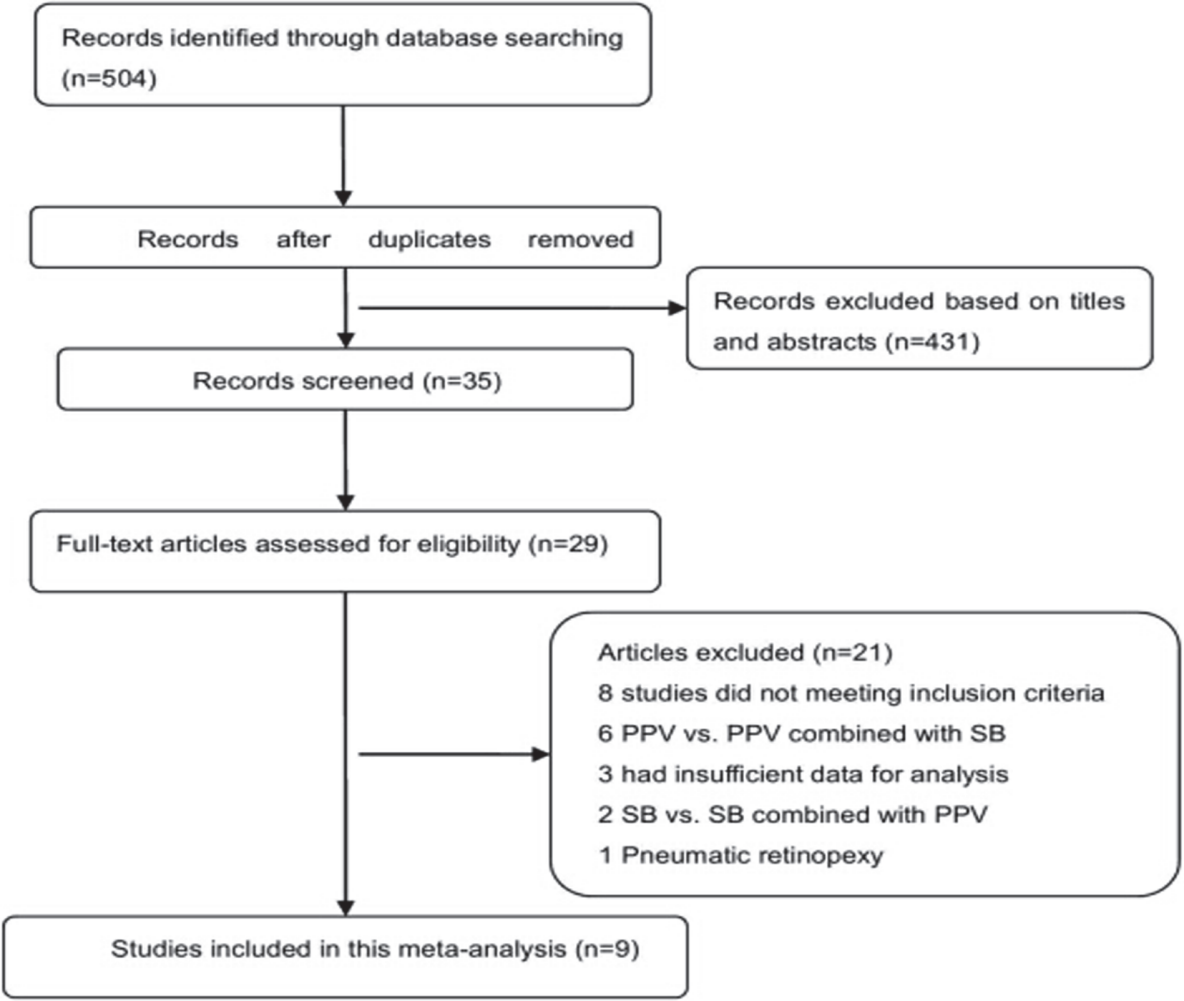
Flow chart of the articles selection process.

An electronic search was conducted for relevant available articles published in English in three databases: the PubMed database (National Center for Biotechnology Information, NCBI), ISI Web of Knowledge and the Cochrane Central Register of Controlled Trials up to March 2014. Using the terms “retinal detachment,” OR “retina detachment,” “scleral buckling,” OR “buckling, scleral” “vitrectomy,” OR “vitrectomies”. The search was limited to human studies. A manual cross-reference search of bibliographies was conducted to identify additional studies. All relevant articles identified through the search were scanned based on the title, keywords, and abstract by two investigators. All studies and analyses were in accordance with the meta-analysis (PRISMA) statement[10]. Disagreements were resolved by discussion. The literature selection process is shown in Fig. 1.

### Study inclusion criteria

Studies were included if they were randomized controlled trials (RCTS) or observational studies published in English; patients diagnosed as primary RRD or with PVR grade-B or less; surgical interventions included SB and PPV. The SB procedure involved encircling buckling and/or explants, and cryopexy/diathermy. Selected procedures included external subretinal fluid (SRF) drainage, balanced salt solution injection, and tamponed. The PPV procedure offered a direct approach for the relief of vitreo-retinal traction, removal of media opacities and detection of obscure retinal breaks. Selected procedures included internal SRF drainage, cryopexy, endolaser treatment, and tamponed. Follow-up duration lasted for at least 6 months. For more detailed information, see the PRISMA checklist (S1 File).

### Data extraction

Data extraction was undertaken according to the predesigned data extraction form. Each retrieved data used standardized forms, obtaining information on author, publication year, country of origin, study design, number of SB or PPV treated eyes and inclusion criteria.

### Quality assessment

The risk of bias assessment of RCTs and nonrandomized studies were respectively using the Cochrane Risk of Bias tool[11] (S2 File) and ACROBAT-NRS by Cochrane[12] (S3 File). We found low risk in both of them in quality assessment. All involved studies were screened for quality and relevance. They had to meet the criteria of the case, matched by the patient’s characteristics.

### Outcome measures

The complications include occurred during the operation, on the early or late stage of post-operation. Intra-operative complications contained subretinal hemorrhage, hypotony and iatrogenic breaks; the early stage of post-operative complications included choroidal detachment, residual subretinal fluid (SRF), elevated IOP and epithelia defect; the late post-operative complications involved diplopia/extraocular muscle (EOM) dysfunction, cataract, cystoid macular edema (CME), macular pucker, post-operative PVR and epiretinal membrane. We analyzed the preoperative macula-off or on would influence the choice of surgical procedures or not from five studies [13–17]. As a result, there exist no significant difference (OR, 1.23; 95%CI, 1.00–1.52; p = 0.06).Therefore we did not perform sub-group analysis of the preoperative macula-off or on. It was thought that the lens status (phakic or pseudophakic/aphakic) would not have any influence on the complications[18], thus, this meta-analysis did not subgroup into phakic versus pseudophakic/aphakic.

### Statistical analysis

Review Manager 5.0 software (The Cochrane Collaboration, Copenhagen, Denmark) was used on statistical analyses. Statistical summaries are presented as odds ratios (ORs) with 95% confidence intervals (CIs). For dichotomous outcomes, the OR was calculated. For continuous outcomes data, the means and standard deviations were used to calculate the estimated mean difference (MD). Heterogeneity was assessed using the chi-square test on Cochrane’s Q statistic and by calculating I^2^ [19]. Statistically significant heterogeneity was considered to be present when *p* heterogeneity <0.05 and I^2^>50%. In the presence of substantial heterogeneity (I^2^>50%), a random effect model was adopted as the pooling method. Otherwise, a fixed effect model was used. Subgroup analysis and asymmetry assessment of the funnel plot for evaluating publication biases were not conducted due to the limited numbers of studies involved in the final analysis.

## Result

### Study characteristics

The initial search identified a total of 504 publications from electronic searches, 495 of which were rejected. The selection process for inclusion of reports is outlined in Fig. 1, nine eligible publications with total 2028 eyes including 1086 SB treated and 942 PPV treated were involved in this meta-analysis[3,4,13–16,20–22]. Clinical follow-ups lasted for at least 6 months. The detailed characteristics of the participants in the 9 included articles were given in Table 1. From Table 1, based on the similar pre-operative or post-operative BCVA results and primary or secondary retinal reattachment rate existed between the SB and PPV groups, the basic characteristics between the two groups were match, as a consequence, we can discuss their surgical complications. The outcomes of RCTs and that of observational studies were detailed described in Table 2.

**Table 1:** Characteristics of included studies.

Study/Year	Countries	Study design	No.eyes (SB/PPV)	Mean age (years) (SB/PPV)	Preoperative BCVA(log MAR)(SB/PPV)	Postoperative BCVA(log MAR) (SB/PPV)	Primary reattachment rate (%) (SB/PPV)	Secondary reattachment rate (%) (SB/PPV)
Ahmadieh 2005	Iran	RCT	126/99	64.23±11.34/60.63±13.65	2.21±0.67/2.37±0.46	0.96±0.68/0.96±0.62	68.3/62.6	85.0/92.9
Azad 2007	India	RCT	31/30	36±16/41±15	1.43±0.92/1.73±0.91	0.608±0.36/0.689±0.35	80.6/80.0	100/100
Brazitikos 2005	Greece	RCT	75/75	71.01±8.13/73.01±8.57	1.09±0.46/0.98±0.52	0.40±0.48/0.33±0.32	82.7/94.7	94.7/98.7
Heimann 2007	Germany	RCT	342/339	63.28±11.10/61.60±10.75	1.03±0.72/1.04±0.77	0.38±0.13/0.44±0.50	59.6/67.0	95.3/96.2
Kobashi 2013	Japan	Retrospective Comparative case series	271/271	43.3±17.5/58.0±8.7	NA	NA	93.7/96.3	100/100
Koriyama 2007	Japan	RCT	23/23	59.4±6.6/61.1±7.2	NA	NA	91.3/91.3	100/100
Miki 2001	Japan	Retrospective Comparative case series	138/87	NA	NA	NA	92/92	100/100
Oshima 2000	Japan	Retrospective comparative case series	55/47	54.3±11.4/58.7±12.9	NA	NA	90.9/91.5	100/100
Sharma 2005	India	RCT	25/25	56.8±12.0/58.28±9.14	NA	NA	76/84	100/100

RCT, randomized controlled trial; BCVA, Best Corrected Visual Acuity.

**Table 2:** The outcomes of RCTs and observational studies.

	RCTs					Observational studies				
complications	No.of eyes (SB/PPV)	ORs(95%CI)	p-value		I^2^(%)*	p-value*	No.of eyes (SB/PPV)	ORs(95%CI)	p-value	I^2^(%)*	p-value*
Total intra-operative complications	19/4	3.64(1.47–9.00)		0.005	50	0.09	10/30	0.27(0.13–0.55)	0.0003	79	0.002
subretinal hemorrhage	6/1	4.60(0.75–27.99)		0.10	0	0.43	9/1	4.81(0.83–27.96)	0.08	0	0.52
hypotony	13/0	18.24(2.37–140.44)		0.005	0	0.47	NA	NA	NA	NA	NA
iatrogenic breaks	0/3	0.12(0.01–2.52)		0.17	NA	NA	1/29	0.04(0.01–0.21)	0.0001	0	0.88
Total early post-operative complications	55/29	1.71(1.09–2.67)		0.02	48	0.03	2/1	1.40(0.24–8.30)	0.71	39	0.20
choroidal detachment	22/2	6.62(2.16–20.34)		0.001	0	0.64	2/0	5.04(0.24–105.41)	0.30	15	0.31
residual SRF	11/0	14.71(1.84–117.32)		0.01	0	0.89	NA	NA	NA	NA	NA
elevated IOP	21/24	0.74(0.39–1.37)		0.33	40	0.17	0/1	0.28(0.01–7.02)	0.44	NA	NA
epithelia defect	1/3	0.41(0.06–2.87)		0.37	0	0.84	NA	NA	NA	NA	NA
Total Late post-operative complications	215/259	0.74(0.60–0.91)		0.98	NA	NA	21/41	0.40(0.24–0.67)	0.0005	50	0.04
diplopia/EOM dysfunction	11/2	3.60(1.05–12.33)		0.04	0	0.76	2/0	5.04(0.24–105.41)	0.30	NA	NA
cataract	96/165	0.42(0.31–0.58)		<0.00001	29	0.24	6/32	0.12(0.05–0.28)	<0.00001	80	0.02
CME	15/17	0.67(0.32–1.41)		0.29	0	0.77	2/1	2.01(0.18–22.17)	0.57	NA	NA
macular pucker	26/14	1.60(0.82–3.15)		0.17	0	0.39	1/2	0.50(0.04–5.53)	0.57	NA	NA
post-operative PVR	66/60	1.11(0.76–1.62)		0.59	0	0.42	3/3	0.90(0.18–4.55)	0.90	0	0.93
epiretinal membrane	1/1	0.97(0.06–16.19)		0.98	NA	NA	7/3	1.73(0.44–6.72)	0.43	0	0.36

RCT, randomized controlled trial; SRF, residual subretinal fluid; IOP, intraocular pressure; EOM, extraocular muscle dysfunction; CME, cystoid macular edema; PVR, proliferative vitreoretinopathy.

*, I^2^ and p-value for Heterogeneity.

### Summary results of surgical complications

#### Intra-operative complications

**Fig 2:**
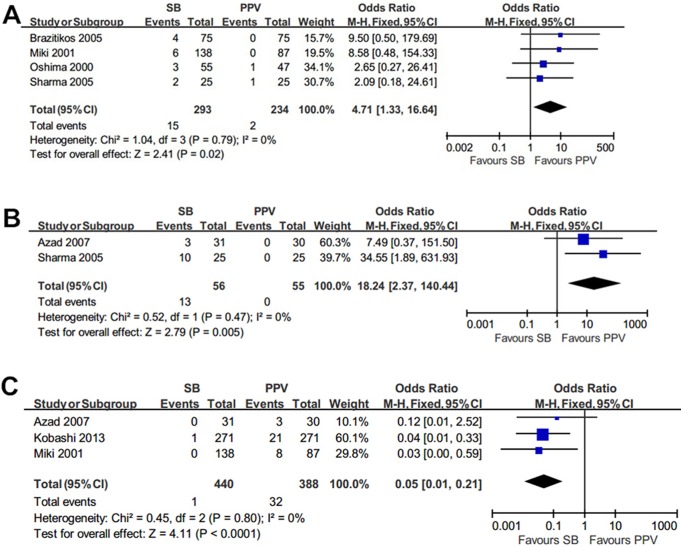
Summary results of intra-operative complications. The forest plot showed the subretinal hemorrhage (A), hypotony (B), iatrogenic breaks (C) along with their associated 95%CIs, comparing SB-treated eyes with PPV-treated eyes.

Subretinal hemorrhage was reported in 15 of 293 eyes (5.1%) in the SB group compared with 2 of 234 eyes (0.9%) in the PPV group. Significantly lower subretinal hemorrhage incidence was in the PPV group (OR = 4.71; 95%CI, 1.33–16.64; *p* = 0.02) (Fig. 2A). No hypotony was found in the PPV group, meanwhile, 13 of 56 eyes (23.2%) occurred in the SB group, OR was 8.24 (95%CI, 2.37–140.44; *p* = 0.005), with no heterogeneity identified (Fig. 2B). Iatrogenic breaks incidence was significant higher in the PPV group than in the SB group, with OR 0.05 (95%CI, 0.01–0.21; *p*<0.0001)(Fig. 2C). No heterogeneity was identified in the intra-operative complication comparisons (*I²* = 0, *p* = 0.79; *p* = 0.49; *p* = 0.80; respectively).

#### Early post-operative complications

**Fig 3:**
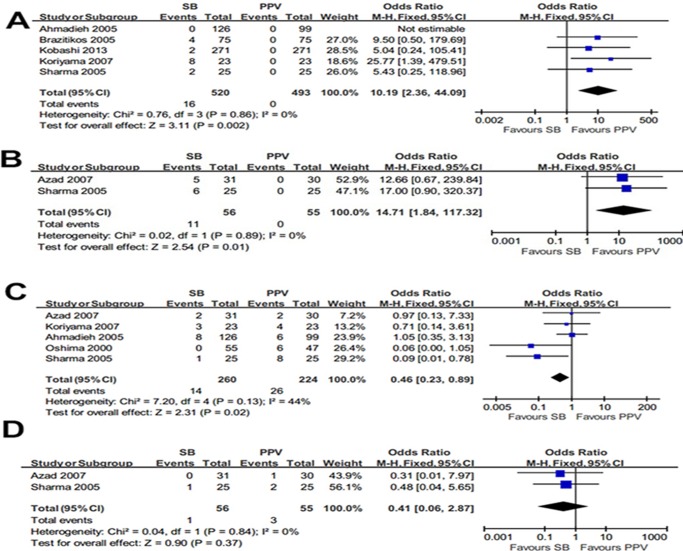
Summary results of early post-operative complications. The forest plot showed the choroidal detachment (A), SRF (B), elevated IOP (C), epithelia defect (D) along with their associated 95%CIs, comparing SB-treated eyes with PPV-treated eyes.

Significantly higher incidence of choroidal detachment was identified with 16 of 520 eyes (3.1%) in the SB group, while it was not found in the PPV group (OR = 10.19; 95%CI, 2.36–44.09; *p* = 0.002) (Fig. 3A). Residual SRF was reported in 11 of 56 eyes in the SB group, while none was found in the PPV group (OR = 14.71; 95%CI, 1.84–117.32; *p* = 0.01) (Fig. 3B). It indicated that these two complications were greatly decreased in the PPV group. Nevertheless, elevated IOP was existed in 14 of 260 eyes (5.4%) in the SB group compared with 26 of 224 eyes (11.6%) in the PPV group which indicated that the odds of elevated IOP is 2.1 times higher in the PPV group than in the SB group (OR = 0.46; 95%CI, 0.23–0.89; *p* = 0.02) (Fig. 3C). No significant difference was demonstrated on the occurrence of epithelia defect compared SB with PPV procedure (OR = 0.41; 95%CI, 0.06–2.87; *p* = 0.37) (Fig. 3D). No or low heterogeneity was identified in the early post-operative complication comparisons (*I²* = 0, *p* = 0.86; *I²* = 0, *p* = 0.89; *I²* = 44%, *p* = 0.13; *I²* = 0, *p* = 0.84; respectively).

#### Late post-operative complications

**Fig 4:**
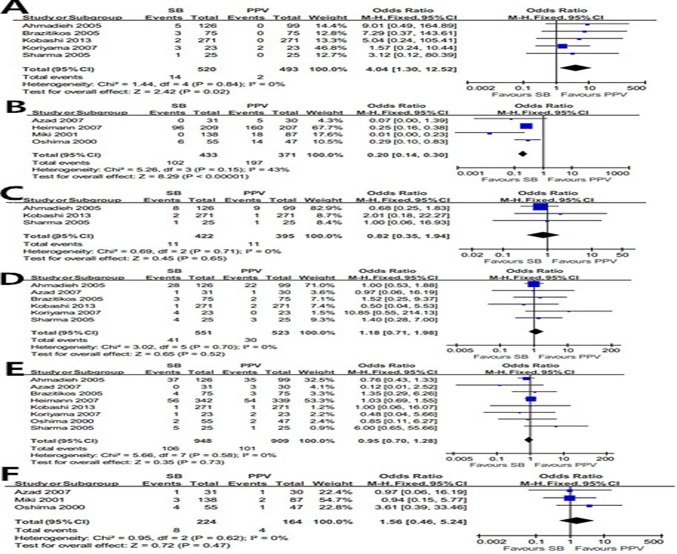
Summary results of late post-operative complications. The forest plot showed the diplopia/EOM dysfunction (A), cataract (B), CME (C), macular pucker (D),post-operative PVR (E), epiretinal membrane (F) along with their associated 95%CIs, comparing SB-treated eyes with PPV-treated eyes.

Diplopia/EOM dysfunction was demonstrated in 14 of 520 eyes (2.7%) in the SB group compared with 2 of 439 eyes (0.5%) in the PPV group. PPV produced significantly less diplopia/EOM dysfunction than SB on primary RRD (OR = 4.04; 95%CI, 1.30–12.52; *p* = 0.02) with no heterogeneity identified (*I²* = 0; *p* = 0.84) (Fig. 4A). Cataract was identified in 102 of 433 eyes (23.6%) in the SB group compared with 197 of 371 eyes (53.1%) in the PPV group. Obviously more cataract cases were derived from PPV management than SB (OR = 0.20; 95%CI, 0.14–0.30; *p*<0.00001) with low heterogeneity (*I²* = 43%; *p* = 0.15) (Fig. 4B). No significant difference was existed in the incidence comparisons of CME (OR = 0.82; 95%CI, 0.35–1.94; *p* = 0.65)(Fig. 4C), macular pucker (OR = 1.18; 95%CI, 0.71–1.98; *p* = 0.52) (Fig. 4D), post-operative PVR (OR = 0.95; 95%CI, 0.70–1.28; *p* = 0.73) (Fig. 4E) and epiretinal membrane (OR = 1.56; 95%CI, 0.46–5.24; *p* = 0.47) (Fig. 4F) between the two groups.

## Discussion

During the RRD operation, significantly lower incidences of subretinal hemorrhage or hypotony were reported in the PPV group. The drainage of subretinal fluid during SB procedure can lead the occurrence of subretinal hemorrhage or hypotony[23], while such technique is no longer applied in the vitrectomy which greatly decreases such complications.

PPV group was demonstrated significantly higher occurrence rate of iatrogenic breaks than SB group. Iatrogenic breaks formation during vitrectomy generally occurs close to the sclera, resulting from vitreous incarceration to the wound and vitreous contraction thereafter, insufficient vitrectomy accompanied by vitreous contraction due to gas tamponade is another possible causative factor[17]. In our opinion, the iatrogenic breaks cannot be regarded as serious complication because it can be timely discovered and patched during the operation. It is presumed that this complication will be solved with the improvement of the instrument innovation and surgeons’ vitrectomy techniques and carefulness.

In the early stage after primary RRD operation, choroidal detachment was identified significantly less in the PPV group which has been reported as one of the most common post-operative complications of SB for repairing primary RRD[24,25]. Choroidal detachment may occur as a result of hypotony in scleral buckling, the risk factors include trauma, particularly the surgical trauma of choroidal perforation, systemic hypertension, glaucoma and high myopia[23,26]. Some surgical procedures such as scleral buckling and puncture used during SB procedure are no longer existed in vitrectomy; moreover, small-gauge transconjunctival instrumentation used in vitrectomy has been reported to reduce surgical complications[27].

More residual SRF was obtained in the SB group. Many factors[28,29] have been suggested to exert an influence on the persistence of SRF after SB surgery, in which, scleral buckle has been observed to affect the subfoveal choroidal blood flow; the hemodynamic change may alter the polarity of retinal pigment epithelium, leading to fluid leakage; moreover, inflammation induced by scleral buckle may contribute to the development of SRF accumulation[30]. It is believed that vitrectomy can effectively decrease such complication. However, vitrectomy is tended to responsible for the significantly post-operative high IOP. No difference was identified in the epithelia defect between the two groups. Frequent fluid–gas exchange in vitrectomy is considered to cause the post-operative high IOP or epithelia defect[31]. The fluid-air or fluid-gas exchanges at the end of operation are adopted to establish an air- or a gas-filled vitreous cavity which facilitates superior wound integrity and therefore decreases the risks of these two complications[32,33].

In the late stage after primary RRD operation, different from previous study[18], significantly less diplopia/EOM dysfunction was reported in the PPV group. Diplopia/EOM dysfunction in SB includes breakdown of an existing fusion weakness, the distortion of the globe by scleral buckles, damage to the rectus muscle insertions by traction sutures, and post-operative tissue swelling. Furthermore, a large or deep buckle used in the case of deeply located or large tears sometimes leads to disturbance in ocular motility[34]. On the contrary, vitrectomy affords a direct approach to retinal breaks and detachment using microsurgical techniques, avoiding injury of the extraocular muscles and buckle infection.

In this meta-analysis, consistent with previous study[18], cataract was considered as the main complication of vitrectomy late after operation. It is probably caused by the removal of the retrolental vitreous with direct gas lens contact[35], preexisting nuclear sclerosis, light toxicity from the operating microscope, intra-operative oxidation of lens proteins, usage of silicone oil or intravitreal gas, intra-operative mechanical trauma or duration of exposure to irrigating solution[36]. In recent years, microincision transconjunctival sutureless vitrectomy become the dominant techniques which has deeply decreased the risk of cataract formation[37].

No significant statistical difference was discovered in the comparison of post-operative PVR which mainly lead to retinal re-detachment. Vitrectomy is suggested as the substitute for SB in the uncomplicated cases for its ability to remove the vitreous traction bands and RPE cells which may cause post-operative PVR[38,39]. However, this meta-analysis did not confirm this advantage of vitrectomy. Chronic inflammation and vitreous traction in some eyes can probably increase the risk of CME, vitrectomy has been shown to have a therapeutic role for CME[40]; nevertheless, no difference existed in the CME incidence between the two groups. No difference was found in the comparisons of other late post-operative complications, including macular pucker and epiretinal membrane.

In this meta-analysis, inconsistent with previous study[18], we reported statistically significant differences in choroidal detachment, elevated IOP or diplopia/EOM dysfunction between the SB and PPV groups. We attributed this to the larger sample size of the current study.

The scleral buckle in SB procedure is left in place permanently and behaves as a foreign body to the eye. Infection or extrusion of the buckle maybe arises even after many years of the surgery. The buckle was reported eroding into the eye, necessitating its removal in some cases[41]. However, such problems are labeled as the phenomenon of SB.

Several limitations of the current meta-analysis could affect the final conclusion. First, the included studies were heterogeneous in terms of study location, population, number of participants and basal condition. Second, the observational studies were prone to bias due to uncontrolled confounding. Third, only studies published in English were included. More data accumulation is necessary.

In summary, this meta-analysis demonstrated that PPV achieved more favorable effects with less intra- or post-operative complications than SB in the management of primary RRD. With the developments of smaller-gauge transconjunctival sutureless vitrectomy techniques, PPV could be considered as potential surgical option on primary RRD.

## Supporting Information

S1 File PRISMA Checklist. (DOC)

S2 File Cochrane Risk of Bias tool for RCTS. (DOC)

S3 File A Cochrane Risk of Bias Assessment Tool: for Nonrandomized Studies of Interventions. (DOC)
