# Supplementary figures and images for: Surgical Complications of Primary Rhegmatogenous Retinal Detachment: A Meta-Analysis
Source: PLoS One. 2015 Mar 3;10(3):e0116493. doi: 10.1371/journal.pone.0116493 (PMC4348461; doi:10.1371/journal.pone.0116493)

Risk of bias graph：


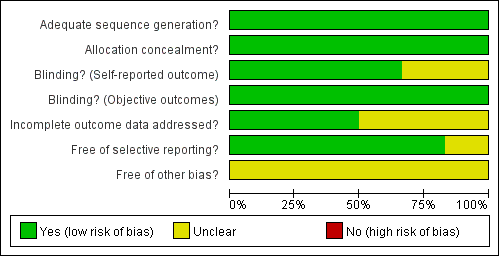


Risk of bias summary：


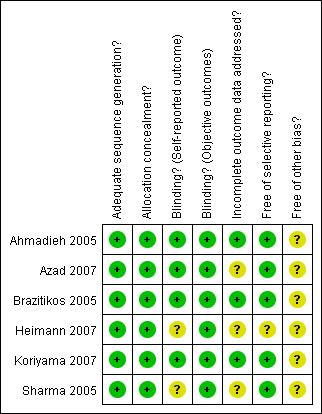

Supplement: S2 File — (DOC) [file pone.0116493.s002.doc]
