# Supplementary material for: Surgical Complications of Primary Rhegmatogenous Retinal Detachment: A Meta-Analysis
Source: PLoS One. 2015 Mar 3;10(3):e0116493. doi: 10.1371/journal.pone.0116493 (PMC4348461; doi:10.1371/journal.pone.0116493)
Supplement: S3 File — (DOC) [file pone.0116493.s003.doc]

## Risk of bias assessment (case-control studies).

| Bias due to confounding | 1.1 Is confounding of the effect of intervention unlikely in this study?  **If Y or PY to 1.1:** the study can be considered to be at low risk of bias due to confounding and no further signalling questions need be considered | **Y** / PY / PN / N | [Description] |
| --- | --- | --- | --- |
|  | **If N or PN to 1.1:** |  |  |
|  | 1.4. Did the authors use an appropriate analysis method that adjusted for all the critically important confounding domains? | NA / Y / PY / PN / N / NI | [Description] |
|  | 1.5. **If Y or PY to 1.4**: Were confounding domains that were adjusted for measured validly and reliably by the variables available in this study? | NA / Y / PY / PN / N / NI | [Description] |
|  | 1.6. Did the authors avoid adjusting for post-intervention variables? | NA / Y / PY / PN / N / NI | [Description] |
|  | **Risk of bias judgement** | **Low** / Moderate / Serious / Critical / NI | [Support for judgement] |
|  | Optional: What is the predicted direction of bias due to confounding? | Favours experimental / Favours comparator / Unpredictable | [Rationale] |
| Bias in selection of participants into the study | 2.4 Were the controls sampled from the population that gave rise to the cases, or using another method that avoids selection bias? | Y / **PY** / PN / N / NI | [Description] |
|  | **Risk of bias judgement** | **Low** / Moderate / Serious / Critical / NI | [Support for judgement] |
|  | Optional: What is the predicted direction of bias due to selection of participants into the study? | Favours experimental / Favours comparator / Towards null /Away from null / Unpredictable | [Rationale] |
| Bias in measurement of interventions | 3.1 Is intervention status well defined? | Y / **PY** / PN / N / NI | [Description] |
|  | 3.2 Was information on intervention status recorded at the time of intervention? | **Y** / PY / PN / N / NI | [Description] |
|  | 3.3 Was information on intervention status unaffected by knowledge of the outcome or risk of the outcome? | Y / **PY** / PN / N / NI | [Description] |
|  | **Risk of bias judgement** | **Low** / Moderate / Serious / Critical / NI | [Support for judgement] |
|  | Optional: What is the predicted direction of bias due to measurement of outcomes or interventions? | Favours experimental / Favours comparator / Towards null /Away from null / Unpredictable | [Rationale] |
| Bias due to departures from intended interventions | 4.1. Were the critical co-interventions balanced across intervention groups? | Y / **PY** / PN / N / NI | [Description] |
|  | 4.2. Were numbers of switches to other interventions low? | Y / PY / **PN** / N / NI | [Description] |
|  | 4.3. Was implementation failure minor? | Y / PY / PN / **N** / NI | [Description] |
|  | **Risk of bias judgement** | Low / **Moderate** / Serious / Critical / NI | [Support for judgement] |
|  | Optional: What is the predicted direction of bias due to departures from the intended interventions? | Favours experimental / Favours comparator / Towards null /Away from null / Unpredictable | [Rationale] |
| Bias due to missing data | 5.1 Was outcome status reasonably complete for those in whom it was sought? | **Y** / PY / PN / N / NI | [Description] |
|  | 5.2 Were data on intervention status reasonably complete? | Y / **PY** / PN / N / NI | [Description] |
|  | 5.3 Are data reasonably complete for other variables in the analysis? | **Y** / PY / PN / N / NI | [Description] |
|  | 5.4 **If N or PN to 5.1, 5.2 or 5.3**: Are the proportion of participants and reasons for missing data similar across cases and controls? | NA / Y / PY / PN / N / NI | [Description] |
|  | 5.5 **If N or PN to 5.1, 5.2 or 5.3**: Were appropriate statistical methods used to account for missing data? | NA / Y / PY / PN / N / NI | [Description] |
|  | **Risk of bias judgement** | **Low** / Moderate / Serious / Critical / NI | [Support for judgement] |
|  | Optional: What is the predicted direction of bias due to missing data? | Favours experimental / Favours comparator / Towards null /Away from null / Unpredictable | [Rationale] |
| Bias in measurement of outcomes | 6.1 Was the definition of case status (and control status, if applicable) based on objective criteria? | **Y** / PY / PN / N / NI | [Description] |
|  | 6.2 Was the definition of case status (and control status, if applicable) applied without knowledge of the intervention received? | **Y** / PY / PN / N / NI | [Description] |
|  | **Risk of bias judgement** | **Low** / Moderate / Serious / Critical / NI | [Support for judgement] |
|  | Optional: What is the predicted direction of bias due to definitions of case and control status? | Favours experimental / Favours comparator / Towards null /Away from null / Unpredictable | [Rationale] |
| Bias in selection of the reported result | Is the reported effect estimate unlikely to be selected, on the basis of the results, from... |  |  |
|  | 7.1. ... multiple *definitions of the intervention*? | Y / **PY** / PN / N / NI | [Description] |
|  | 7.2 ... multiple *analyses* of the intervention-outcome relationship? | Y / **PY** / PN / N / NI | [Description] |
|  | 7.3 ... different *subgroups*? | **Y** / PY / PN / N / NI | [Description] |
|  | **Risk of bias judgement** | **Low** / Moderate / Serious / Critical / NI | [Support for judgement] |
|  | Optional: What is the predicted direction of bias due to selection of the reported result? | Favours experimental / Favours comparator / Towards null /Away from null / Unpredictable | [Rationale] |
| Overall bias | **Risk of bias judgement** | **Low** / Moderate / Serious / Critical / NI | [Support for judgement] |
|  | Optional: What is the overall predicted direction of bias? | Favours experimental / Favours comparator / Towards null /Away from null / Unpredictable | [Rationale] |
